# Fracture strength and ribbond fibers: In vitro analysis of mod restorations

**DOI:** 10.4317/jced.60334

**Published:** 2023-04-01

**Authors:** Francesca Zotti, Jie Hu, Alessandro Zangani, Massimo Albanese, Corrado Paganelli

**Affiliations:** 1 Researcher, Department of Surgical Sciences, Paediatrics and Gynecology, University of Verona, 37134 Verona, Italy (IT); 2 M.D., Private Practice, 37134, Verona, Italy; 3 PhD Student, Department of Surgical Sciences, Paediatrics and Gynecology, University of Verona, 37134 Verona, Italy (IT); 4 Full professor, Department of Surgical Sciences, Paediatrics and Gynecology, University of Verona, 37134 Verona, Italy (IT); 5 Full professor, Department of Medical and Surgical Specialties, Radiological Sciences and Public Health, Dental School, University of Brescia, Brescia, Italy

## Abstract

**Background:**

Ribbond fibers are supposed to be a reinforcing material in restoration of compromised teeth. This study aims to compare MOD restorations with and without Ribbond Fiber in terms of fracture strength under axial loading; to identify the minimum depth of MOD cavities to use Ribbond Fiber (to improve the fracture strength under axial load.

**Material and Methods:**

20 upper and lower molars extracted intact were used for the experiment. The teeth were prepared with 2 types of cavities and then divided into 4 groups: 1) 5 mm deep MOD cavities with residual interaxial dentin restored without Ribbond; 2) 5 mm deep MOD cavities with residual interaxial dentin restored with Ribbond; 3) 5 mm deep MOD cavities without residual interaxial dentin, restored without Ribbond; 4) 5 mm deep MOD cavities without residual interaxial dentin restored with Ribbond. The restored teeth were then subjected to thermal cycling and their fracture strength was evaluated using an Instron device. The Mann-Whitney statistical test was used to compare fracture strength among groups. Finally, a descriptive analysis of the verified fractures was performed.

**Results:**

There was a statistically significant difference between groups 1 and 2 (*P* = 0.0090) in the loading force required for a fracture. In contrast, there was no statistically significant difference between groups 3 and 4 (*P* = 0.7540). Groups 1 and 2 had the fewest non-restorable fractures, in contrast to groups 3 and 4.

**Conclusions:**

Ribbond fiber application in MOD cavities seems to be more effective in terms of strengthening where cavities have interaxial dentinal tissue.

** Key words:**Ribbond fibers, fracture strenght, direct dental restorations.

## Introduction

In determining prognosis in restorative dentistry, the amount of remaining healthy tooth tissue plays a critical role. Several structural factors act as predictors of the prognosis of restorations: the interaxial dentin, the roof of the pulp chamber, the marginal ridges and the residual amount of cusps (1). Among them, the interaxial dentin and marginal ridges have the greatest influence, and it is important to know that all these factors are closely related. It follows that the fracture resistance of restorations depends on the morphology of the cavity and that MOD (mesial-occlusal-distal) is the most susceptible to fracture. A Class I cavity results in a 20% loss of stiffness, which increases to 40% if one of the marginal ridges is lost and to 60% if MOD is lost (2). Regarding fracture strength, the literature indicates 3000 N required to fracture healthy molars (3), between 1200 and 700 N to fracture Class I molars, depending on the size of the cavity, and between 1100 and 600 N for Class II. MOD Molar cavities have a fracture strength of 1000-500 N (4,5). These values increase when teeth are restored, restored Class I molars reach a fracture strength between 1000 and 1900 N (6), and MOD restored molars have a fracture strength of 1600 N (4).

All these values are, of course, dependent on the amount of remaining tooth tissue, the restoration techniques and the restorative materials used (7). In addition to the well-known restorative materials, such as flowable resins and composites, whose performance in terms of mechanical, esthetic and adhesive properties has been extensively studied, fiber-reinforced composites have appeared on the market in recent years. These materials are composite resins reinforced by the use of fiber materials such as glass fibers, carbon fibers, Kevlar or Vectran fibers and polyethylene fibers in the cavity, which significantly improves the mechanical properties of the resins. Polyethylene fibers are most commonly used in restorative dentistry because they increase the flexural strength, stress resistance, and modulus of elasticity of composite resins while providing good esthetic performance because they are barely visible when immersed in a resin matrix. One of the most commonly used of these materials is Ribbond Fiber (RF), which consists of woven fibers of high-molecular-weight polyethylene. It has a high coefficient of elasticity (117 GPa) and thus high resistance to elongation and deformation, as well as high tensile strength (3 GPa), which allows it to adapt to the tooth cavity and morphology. It undergoes a cold plasma treatment that enables it to absorb water, reduce surface tension and provide improved chemical adhesion to composites (8).

Ribbond fiber is commonly used in various fields of dentistry, but there are no sound results in the literature on the application of Ribbond fiber in the restoration of vital teeth.

The aims of this *in vitro* study were: i) to compare the fracture strength of MOD restorations fabricated with and without Ribbond fiber under axial loading; ii) to determine the minimum size of MOD cavities in which the use of Ribbond fiber is useful in terms of improving fracture strength.

## Material and Methods

Twenty undamaged extracted maxillary and mandibular molars were used for this *in vitro* study. The sample included non-carious and cavitated teeth of classes I, II and MOD. Pulp-damaged or non-vital teeth were excluded from the selection, as were radicular cavitated elements.

All teeth were cleaned and disinfected with a 0.20 chlorhexidine solution for 60 seconds, washed, and kept in physiological solution for a period not exceeding 3 months.

The teeth were divided into 4 groups and prepared as follows:

Groups 1 and 2: 10 molars with MOD cavities in different sizes:

- Occlusal isthmus in vestibular-lingual direction of 3 mm and 3 mm high with 1 mm cylindrical diamond bur;

- Interproximal box with occlusal-apical height of 5 mm, vestibular-lingual width of 4 mm and mesio-distal depth of 3 mm;

- Remaining thickness of the vestibular and lingual walls of at least 2 mm;

- Beveling of the edges and removal of unsupported enamel prisms with a 1 mm diamond ball bur.

Groups 3 and 4: 10 molars with a MOD cavity of 5 mm height and width leaving a maximum thickness of 2/3 mm at the level of the vestibular and lingual walls were prepared with 1 mm cylindrical diamond burs. The edges were then beveled and the unsupported enamel prisms were removed with a 1 mm spherical diamond bur.

Groups 2 and 4 were restored adding Ribbond fibers (Ribbond, Ribbond Inc., Seattle, WA, USA) and those of the groups 1 and 3 without.

Adhesive procedures were performed with Tokuyama Etching Gel HV (Tokuyama Dental Corp., Japan) and Tokuyama EE Bond (Tokuyama Dental Corp., Japan). Restorations were fabricated with multiple increments of Ceram.x Spectra ST (Dentsply Sirona, Ballantyne Corporate Pl, Charlotte, NC, USA) and flowable composite (Estelite Flow Quick - Tokuyama Dental Corp, Japan).

For restorations with fibers, a 2-mm-wide and 8-mm-long strip of polyethylene fibers was immersed in a 1-mm-thick layer of flowable composite at the base of the cavity, in a mesial-distal orientation and as close as possible to the tooth tissue to minimize the thickness of the flowable resin used. Light curing was performed according to the manufacturer’s instructions.

Finishing and polishing were routinely performed.

All restored specimens were therefore thermocycled using the standard ISO TR 11450 (1994): 500 cycles of 30’’ each in water with temperature variations between 5±2°C and 55±2°C. The time was calculated with a digital chronometer and completed in 4 hours and 10 minutes.

For the 30’’ cycle at 55±2°C, an immersion thermostat (Julabo MP -5 Heating Circulator, JULABO GmbH, Germany) was used for temperature control, while the 30’’ cycles at 5±2°C were performed in an ice bath with constant temperature recording.

**Figure 1:**
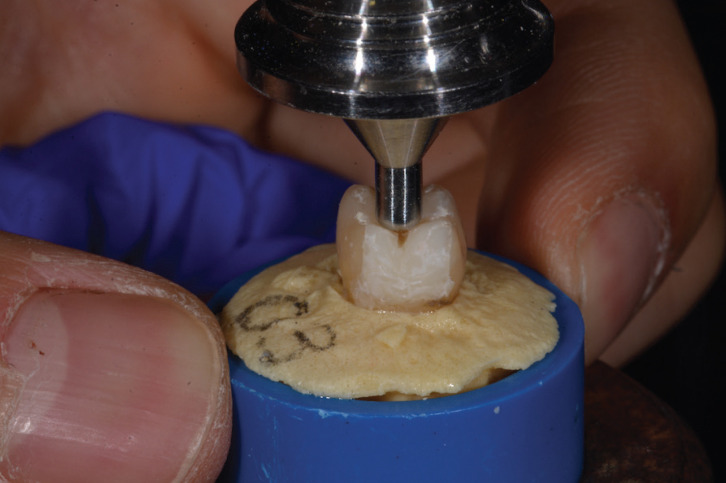
Custom made insert for Instron.

The specimens were placed in PVC boxes with a diameter of 20 mm and a height of 20 mm at the CEJ (cement- enamel junction) using a self-curing acrylic resin (SR Ivolen Kit - Ivoclar Vivadent, Liechtenstein). The boxes were placed on the platform of the 2000-N load cell Instron Machine (Instron 5848, Norwood, Massachusetts USA) to test the fracture strength under vertical axial loads. To achieve closer contact between the fossa-cusps and the Instron tip, a special insert for the machine with a spherical tip of 3 mm was designed and custom made (Fig. 1).

The machine was set to move within a predetermined vertical range of 2.5 mm in 90 seconds, with vertical loading of the occlusal surface at a rate of 0.028 mm/s.

**Figure 2:**
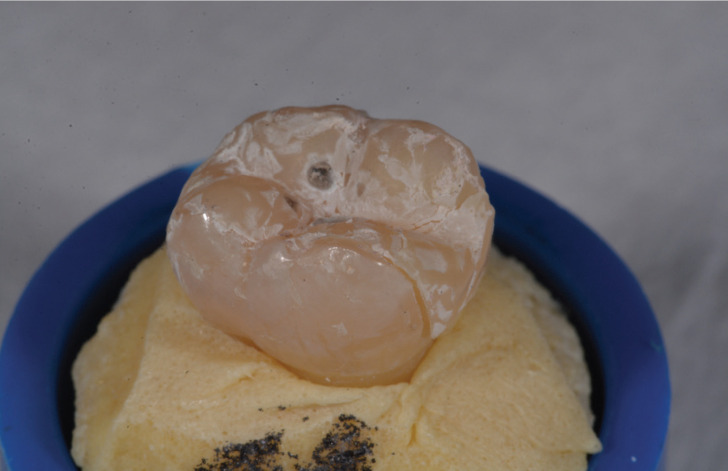
Fracture of the composite resin.

**Figure 3:**
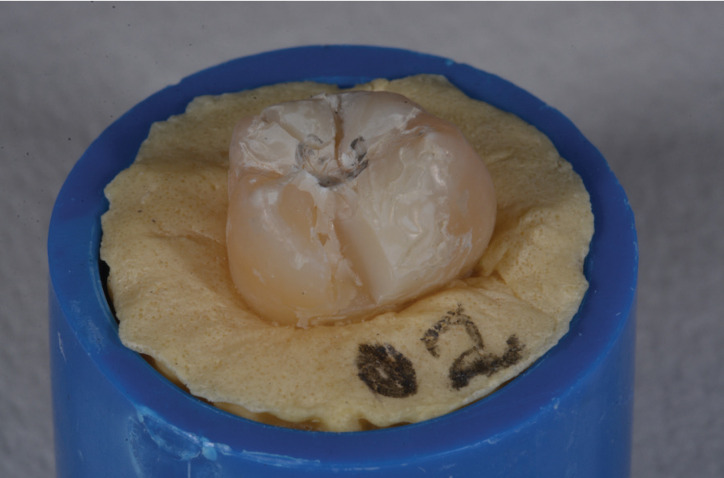
Fracture of one or more cusps.

**Figure 4:**
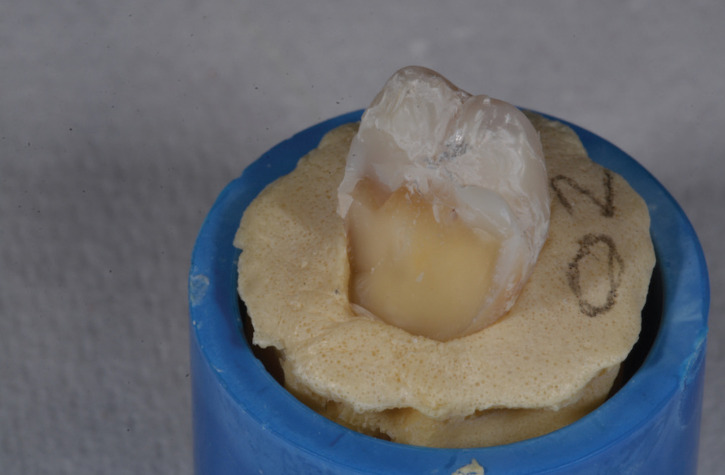
Fracture extending to the CEJ or deeper.

The following data were recorded:

- Load (Newton): Impressed force required to fracture the teeth during the test;

- Vertical area (mm): area in which the fracture occurs;

- Pattern of fracture:

1) Fracture of the composite resin (Fig. 2)

2) Fracture of one or more cusps (Fig. 3)

3) Fracture extending to the CEJ or deeper (Fig. 4)

The Mann-Whitney test was performed to analyze the statistical differences between group 1 (3 mm cavity without Ribbond fiber restorations) and group 2 (3 mm cavity with Ribbond fiber restorations) and between group 3 (5 mm cavity without Ribbond fiber restorations) and group 4 (5 mm cavity with Ribbond fiber restorations) in terms of the stress required for fractures.

Descriptive analysis was performed to evaluate the vertical area (mm) required for fracture and the patterns of the different fractures.

Statistical tests were performed using STATA16 software (StataCorp, 1985, California, USA), and the significance level was set at 0.05.

## Results

Groups were divided into G1 (3 mm MOD no fiber), G2 (3 mm MOD with fiber), G3 (5 mm MOD no fiber) and G4 (5 mm MOD with fiber). Each group consisted of 5 samples, a total of 20 teeth were analysed.

The Mann-Whitney test showed statistically significant differences between G1 and G2 (*P*=0.009) in terms of the stress required for fracture. Between groups 3 and 4 no statistically significant differences were highlighted (*P* = 0.7540).

In G1 the 80% of fractures detected was found to be restorable by a conservative intervention, the same for the G2 in which 80% of the samples was restorable and the fractures detected were visible just in the thickness of the composite resin.

In G3 restorable fractures were the 60% as well as in the G4. G3 and G4 showed the highest number of not restorable fractures.

Results are clearly exposed in  Table 1 and  Table 2.

**Table 1:**
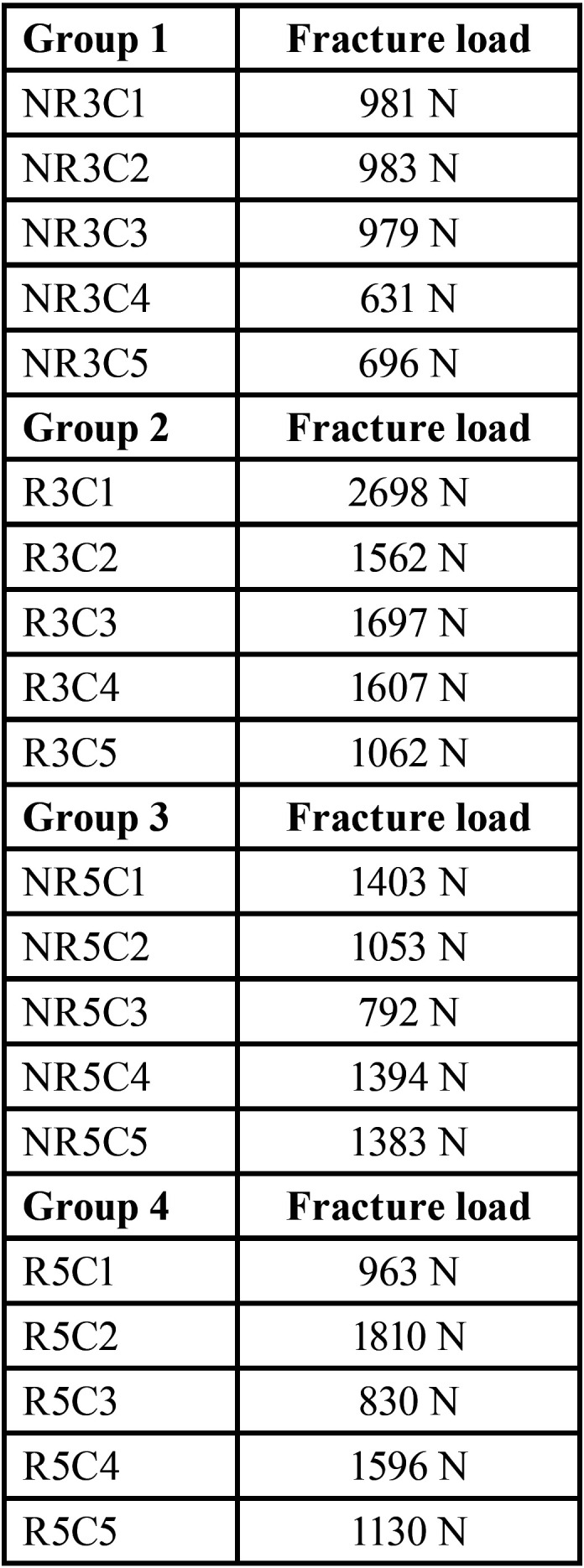
Impressed load required to fracture the teeth for each group.

**Table 2:**
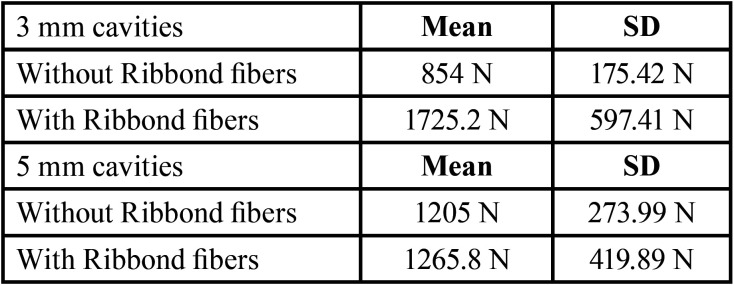
Mean value of loads required to fracture the teeth for each group.

## Discussion

The purpose of this study was to compare and determine under which conditions Ribbond Fiber (Ribbond Inc., Seattle, WA, USA) is best suited to improve the fracture strength of molars with two different types of cavities MOD.

The limited data in the literature indicate that Ribbond seems to be able to improve both the flexural and fracture strength of composite restorations. Due to its network-like structure and multiple interwoven fibers of high molecular weight polyethylene, it is capable of slowing the propagation of cracks in the event of fracture and better distributing the forces exerted on the reconstructed tooth (9). The specimens used are molars, as they are the elements that have the greatest degree of cusp flexion under load in relation to the degree of residual tooth structure, with MOD cavities (10).

Very important is the fact that the cavities were prepared taking into account the pulp cavity, thus simulating a real conservative treatment without imitating an endodontically treated tooth. This aspect is particularly noteworthy because there are few data in the literature evaluating MOD cavities prepared on teeth without endodontic treatment and without involving the pulp chamber roof.

Teeth are constantly subjected to stress during normal oral functions. Prolonged and cyclic occlusal loading may result in cusp displacement (11). In this study, specimens were subjected to continuous loading, whereas cyclic loading was not investigated; this could be a limitation.

In order to proceed with the experimental part of this study, it was necessary to develop a special insert to perform the tests using the Instron (Instron 5848, Norwood, Massachusetts USA). This was necessary because the preliminary tests with test specimens did not give satisfactory results because the inserts supplied by the manufacturer were not able to break the tooth elements except at forces above the normal masticatory load. We assumed that this was due to the shape of the insert used in the tests, which was not able to contact the masticatory fossa and come into close contact with the cusps, which is ineviTable in a functional masticatory process. In fact, the insert provided had a smooth, circular surface with a diameter of 1.5 cm, which did not allow optimal contact with the cusps and pits of the teeth. In the literature, inserts with a spherical surface and a much smaller diameter on the order of 5 mm are commonly used. In literature studies by King and colleagues (12-14), a fracture strength of 2500 to 3500 N was found in healthy mandibular molars using a spherical insert with a diameter of 5.6 mm. In their study, Watts and colleagues (13) investigated fracture resistance using two spherical inserts with different diameters, one 4 mm and one 8 mm. With the 4-mm spherical insert, they determined a fracture toughness of 2740 N, while with the 8-mm insert, they found a fracture toughness of approximately 4000 N. From this, they concluded in their study that the difference in strength is directly related to the diameter of the insert used; the larger the diameter, the higher the value of fracture toughness. This is consistent with the observations from tests conducted prior to our actual experiments. Therefore, in the absence of a suiTable insert for the planned tests, it was decided to design and have manufactured a special spherical insert with a diameter of 3 mm for the Instron (Instron 5848, Norwood, Massachusetts, USA), which proved to be the most suiTable size to obtain maximum contact with the cusps and pits of the specimens.

The values for fracture toughness in N obtained in the study are in agreement with the data observed in the literature for axially loaded molars (15). The values between the different studies in the literature can vary greatly depending on the materials used, the reconstruction technique, the experience of the dentist, whether or not the elements were thermocycled, and the preservation and integrity of the test specimens. In addition, as mentioned above, much depends on the type of insert used and especially on the size of the insert. Finally, results may vary greatly from those reported in the literature due to different cavity configurations.

The literature indicates that fractures rarely occur along the CEJ (15,16) because it is an interface that can act as a shield for the propagation of fracture edges. Therefore, the CEJ should be preserved as much as possible during cavity preparation. According to Bechtle and colleagues (16), fracture arrest occurs only when microcracks reach the CEJ starting from the enamel part of the tooth. If the fracture originates from the dentin part, the tooth may fracture completely after a certain amount of plastic and elastic deformation.

Regardless of how the tooth cavity is prepared, any loss of tooth tissue results in a significant weakening of the tooth structure (5,17).

The first objective of this study was to compare restorations of the MOD type restored with and without Ribbond Fiber (Ribbond Inc., Seattle, WA, USA) to determine if there were differences in fracture toughness in Newtons under axial loading. Statistical analysis showed that there was a statistically significant difference (*P* = 0.0090) between specimens in groups 1 (3-mm cavity restored without RF) and 2 (3-mm cavity restored with RF) with MOD cavities in which an occlusal isthmus of dentin remained above the roof of the pulp chamber. However, no statistically significant difference (*P* = 0.7540) was found between the specimens in groups 3 (5-mm cavity restored without RF) and 4 (5-mm cavity restored with RF). From these results, it can be concluded that Ribbond Fiber is indeed able to strengthen the tooth structure when there is enough interaxial residual dentin to support the tooth structure. In contrast, the complete loss of interaxial dentin results in a loss of fracture strength of the tooth, which does not seem to be influenced by the presence or absence of RF.

The minor influence of RF in deeper MOD cavities without interaxial dentin could be due to the fact that the placement of such a material beyond a certain depth no longer allows it to exert its property of dissipating loading forces.

The second objective of this study was to determine the depth at which the fibers should be placed in order to take full advantage of their properties. Despite the small number of samples in this study, it can be seen that in MOD cavities the maximum depth at which RF ceases to exert its function is 3 mm. This is because there is a significant difference between the use of fibers and non-use between groups 1 and 2. In fact, in group 2, an interaxial dentin portion of about 2 mm was left, which allowed some of the polyethylene material to be in a shallower part of the prepared cavity. In contrast, in groups 3 and 4 this dentin portion was not left, as the entire cavity was prepared with the same depth of 5 mm and thus the entire fiber strip was on the cavity floor, at the same height.

The high standard deviation value of the breaking load in this study, which was also observed by Watts and colleagues in their study (18), is typical for mechanical tests on anatomically irregular specimens, in this case the occlusal surface of molars.

Vertical loading forces generate lateral forces against the MOD cavity walls, which then create a tensile force on the roof of the pulp chamber that may prove responsible for the initial cracking of the residual walls. Given the lack of hardness of composites, irreparable fracture is more likely to occur when a structurally compromised tooth is restored with composite alone (10,19,20). In fact, groups 3 and 4 had the highest number of irreparable fractures beyond the CEJ in both specimens restored with and without RF. As you can see, the lower the residual tooth structure, the lower the ability to resist the applied forces. These forces do not seem to be redistributed and partially absorbed when RF is placed at the base of cavities with a depth greater than or equal to 5 mm. This is consistent with everything that has been said so far. Therefore, the use of Ribbond fibers in cavities of this size does not seem to be able to change the prognosis of the tooth if the intention is to use it as a base for the cavity to be reconstructed. A possible solution to this problem would be to place the RF strip at the level of the coronal third of the tooth when part of the cavity has already been reconstructed, so that the properties of the material can be exploited. Alternatively, Ribbond fibers could not be used as a cavity foundation, otherwise could be bonded directly to the MOD cavity walls to strengthen them. Instead, groups 1 and 2 each had only one irreparable fracture, with the second group having mainly fractures limited to the restoration. This can be attributed to the ability of Ribbond Fiber to relieve axial loading forces by reducing wall stresses.

## References

[1] Goel VK, Khera SC, Gurusami S, Chen RC (1992). Effect of cavity depth on stresses in a restored tooth. J Prosthet Dent.

[2] Reeh ES, Messer HH, Douglas WH (1989). Reduction in tooth stiffness as a result of endodontic and restorative procedures. J Endod.

[3] Fráter M, Forster A, Keresztúri M, Braunitzer G, Nagy K (2014). In vitro fracture resistance of molar teeth restored with a short fibre-reinforced composite material. J Dent.

[4] Ciavoi G, Mărgărit R, Todor L, Bodnar D, Dina MN, Tărlungeanu DI (2021). Base Materials' Influence on Fracture Resistance of Molars with MOD Cavities. Materials (Basel).

[5] Mondelli J, Steagall L, Ishikiriama A, de Lima Navarro MF, Soares FB (1980). Fracture strength of human teeth with cavity preparations. J Prosthet Dent.

[6] Leyton BS, Rached RN, Ignácio SA, Souza EM (2022). Fracture strength of extended class I composite restorations with different restorative techniques. Odontology.

[7] Hervás-García A, Martínez-Lozano MA, Cabanes-Vila J, Barjau-Escribano A, Fos-Galve P (2006). Composite resins. A review of the materials and clinical indications. Med Oral Patol Oral Cir Bucal.

[8] Tuloglu N, Bayrak S, Tunc ES (2009). Different clinical applications of bondable reinforcement ribbond in pediatric dentistry. Eur J Dent.

[9] Deliperi S, Alleman D, Rudo D (2017). Stress-reduced Direct Composites for the Restoration of Structurally Compromised Teeth: Fiber Design According to the "Wallpapering" Technique. Oper Dent.

[10] Belli S, N Dönmez N, Eskitaşcioğlu G (2006). The effect of c-factor and flowable resin or fiber use at the interface on microtensile bond strength to dentin. J Adhes Dent.

[11] Jantarat J, Palamara JE, Messer HH (2001). An investigation of cuspal deformation and delayed recovery after occlusal loading. J Dent.

[12] Re GJ, Norling BK, Draheim RN (1982). Fracture resistance of lower molars with varying faciocclusolingual amalgam restorations. J Prosthet Dent.

[13] Re GJ, Draheim RN, Norling BK (1981). Fracture resistance of mandibular molars with occlusal class I amalgam preparations. J Am Dent Assoc.

[14] Re GJ, Norling BK (1981). Fracturing molars with axial forces. J Dent Res.

[15] Xu HH, Smith DT, Jahanmir S, Romberg E, Kelly JR, Thompson VP (1998). Indentation damage and mechanical properties of human enamel and dentin. J Dent Res.

[16] Bechtle S, Fett T, Rizzi G, Habelitz S, Klocke A, Schneider GA (2010). Crack arrest within teeth at the dentinoenamel junction caused by elastic modulus mismatch. Biomaterials.

[17] Larson TD, Douglas WH, Geistfeld RE (1981). Effect of prepared cavities on the strength of teeth. Oper Dent.

[18] Watts DC, el Mowafy OM, Grant AA (1987). Fracture resistance of lower molars with Class 1 composite and amalgam restorations. Dent Mater.

[19] Akman S, Akman M, Eskitascioglu G, Belli S (2011). Influence of several fibre-reinforced composite restoration techniques on cusp movement and fracture strength of molar teeth. Int Endod J.

[20] Belli S, Erdemir A, Ozcopur M, Eskitascioglu G (2005). The effect of fibre insertion on fracture resistance of root filled molar teeth with MOD preparations restored with composite. Int Endod J.

